# Evaluation of Whole Genome Sequencing-Based Predictions of Antimicrobial Resistance to TB First Line Agents: A Lesson from 5 Years of Data

**DOI:** 10.3390/ijms25116245

**Published:** 2024-06-06

**Authors:** Meenu Kaushal Sharma, Michael Stobart, Pierre-Marie Akochy, Heather Adam, Debra Janella, Melissa Rabb, Mohey Alawa, Inna Sekirov, Gregory J. Tyrrell, Hafid Soualhine

**Affiliations:** 1 National Reference Centre for Mycobacteriology, National Microbiology Laboratory, Public Health Agency of Canada, Winnipeg, MB R3E 3R2, Canadamichael.stobart@phac-aspc.gc.ca (M.S.);; 2 Department of Medical Microbiology & Infectious Diseases, University of Manitoba, Winnipeg, MB R3E 0J9, Canada; hadam@sharedhealthmb.ca; 3 Laboratoire de Santé Publique du Québec-Institut National de Santé Publique du Québec, Sainte-Anne-de-Bellevue, QC H9X 3R5, Canada; 4 Diagnostic Services, Shared Health, Winnipeg, MB R3C 3H8, Canada; 5 Regina Qu’Appelle Health Region, Regina, SK S4T 1A5, Canada; mohey.alawa@saskhealthauthority.ca; 6 Public Health Laboratory, B.C. Centre for Disease Control, Vancouver, BC V5Z 4R4, Canada; inna.sekirov@bccdc.ca; 7 Division of Diagnostic and Applied Microbiology, Department of Laboratory Medicine and Pathology, University of Alberta, Edmonton, AB T6G 2J2, Canada; 8 Alberta Precision Laboratories Public Health, Edmonton, AB T6G 2J2, Canada

**Keywords:** whole genome sequencing, *Mycobacterium tuberculosis*, tuberculosis, identification, antimicrobial resistance

## Abstract

Phenotypic susceptibility testing of the *Mycobacterium tuberculosis* complex (MTBC) isolate requires culture growth, which can delay rapid detection of resistant cases. Whole genome sequencing (WGS) and data analysis pipelines can assist in predicting resistance to antimicrobials used in the treatment of tuberculosis (TB). This study compared phenotypic susceptibility testing results and WGS-based predictions of antimicrobial resistance (AMR) to four first-line antimicrobials—isoniazid, rifampin, ethambutol, and pyrazinamide—for MTBC isolates tested between the years 2018–2022. For this 5-year retrospective analysis, the WGS sensitivity for predicting resistance for isoniazid, rifampin, ethambutol, and pyrazinamide using Mykrobe was 86.7%, 100.0%, 100.0%, and 47.8%, respectively, and the specificity was 99.4%, 99.5%, 98.7%, and 99.9%, respectively. The predictive values improved slightly using Mykrobe corrections applied using TB Profiler, i.e., the WGS sensitivity for isoniazid, rifampin, ethambutol, and pyrazinamide was 92.31%, 100%, 100%, and 57.78%, respectively, and the specificity was 99.63%. 99.45%, 98.93%, and 99.93%, respectively. The utilization of WGS-based testing addresses concerns regarding test turnaround time and enables analysis for MTBC member identification, antimicrobial resistance prediction, detection of mixed cultures, and strain genotyping, all through a single laboratory test. WGS enables rapid resistance detection compared to traditional phenotypic susceptibility testing methods using the WHO TB mutation catalog, providing an insight into lesser-known mutations, which should be added to prediction databases as high-confidence mutations are recognized. The WGS-based methods can support TB elimination efforts in Canada and globally by ensuring the early start of appropriate treatment, rapidly limiting the spread of TB outbreaks.

## 1. Introduction

The consequences of antimicrobial resistance in the clinical management of infectious diseases are substantial. There are four antimicrobials classified as first-line agents recommended for *Mycobacterium tuberculosis* complex (MTBC) treatment: isoniazid, rifampin, ethambutol, and pyrazinamide [1,2]. Mono-resistant TB is defined as resistance to any one of the first-line agents; resistance to rifampin and isoniazid is considered as multidrug resistance (MDR); pre-extensively drug resistance (pre-XDR) is described as MDR or rifampin-resistant (RR) TB that also exhibits resistance to a fluoroquinolone; while extensively drug resistant (XDR) is defined as MDR/RR, resistance to fluoroquinolone and a Group A drug (either levofloxacin, moxifloxacin, bedaquiline, or linezolid) [3,4]. The challenge of eliminating TB is compounded by the emergence of antimicrobial resistance, which hinders eradication efforts due to requirements for alternative treatment regimens and delays in the initiation of effective treatment [1,2,5]. The rapid and accurate identification of antimicrobial resistance in MTBC is essential to the initiation of early, effective treatment, which can reduce the duration of overall treatment and incurred medical expenses, thus enhancing the management of disease progression and control [6,7,8,9,10].

According to the Global Report from the World Health Organization (WHO), the global incidence of TB in 2022 was 133 per 100,000 people, with an estimated 10.6 million newly diagnosed cases and 1.6 million deaths due to TB [4]. Two-thirds of the total cases originated from eight countries: China, India, Indonesia, Pakistan, the Philippines, Nigeria, Bangladesh, and the Democratic Republic of the Congo [4,11]. In Canada, the TB incidence rate for 2021 was 4.8 active TB cases per 100,000 people, with this rate being 12.3 for persons born outside Canada, 0.3 for non-Indigenous Canadian born, 135 for Inuit, 16.1 for First Nation, and 2.1 for Métis persons [7]. From culture positive cases, the proportion of *M. tuberculosis* susceptible to all agents was 90.1%, with mono-resistance at 8.5% and MDR at 1%; and XDR TB was seen infrequently, at 0.1% in 2021 [7]. Of all mono-resistant TB cases, 6.6% were isoniazid-resistant, 0.3% were rifampin-resistant, and 1.6% were pyrazinamide-resistant, with ethambutol mono-resistance being a rare occurrence [7].

Whole genome sequencing (WGS) offers an advantage of providing high-resolution sequence data that may be used for various downstream analyses, including species identification, resistance prediction, outbreak investigation, identification of contamination events, and routine genotyping [12,13,14,15,16,17,18]. The genetic mutations and indels conferring resistance to all first- and second-line agents are extensive and continue to expand [19,20,21,22,23,24]. With increasing understanding of mutations conferring resistance to antimicrobials, it is key that the databases used for predictions are maintained and updated regularly to reflect the most current high-confidence mutations at the time. In this study, the concordance of first-line phenotypic susceptibility testing data and WGS-based prediction of resistance/susceptibility to first line agents was evaluated retrospectively for MTBC isolates tested at the National Reference Center for Mycobacteriology (NRCM) from 2018 to 2022. The additional aim of this study was to evaluate parameters affecting WGS performance, i.e., novel mutations, sequence Quality control (QC) issues, and limitations of the Mykrobe, TB profiler, and BioHansel databases and pipelines utilized in the analyses at the time of reporting. 

## 2. Results

There were a total of 1510 MTBC isolates that met the study criteria for WGS sequence quality. The number of mapped reads varied between 452,251 to 8,473,100, averaging 1,826,456 bp, with a median of 1,632,334 bp. The range of bases meeting the threshold was from 35.19% to 99.95%, with a mean of 94.05% and a median of 97.87%. The average base coverage ranged from 30× to 564×, with a mean of 82× and a median of 73.22×. The number of non-covered bases ranged from 0 to 170,667 bp, averaging 20,659 bp, with a median of 15,882 bp.

The BioHansel pipeline was used to identify all MTBC isolates. A total of 1411 (93.4%) were identified as *M. tuberculosis*, 46 (3%) as *M. orygis*, 19 (1.3%) as *M. bovis* BCG, 16 (1.1%) as *M. africanum*, 10 (0.7%) as *M. bovis*, 2 (0.1%) as *M. canettii*, and 6 (0.4%) failed to be identified by the pipeline. These six were subjected to a WGS SNP-based identification scheme [25], which identified two as *M. bovis*, one each as *M. tuberculosis* and *M. africanum*, and two were identified as *M. tuberculosis* L4 lineage, with novel mutations C1194T and G1292C in the *gyrB* gene.

The sensitivity, specificity, positive predictive value (PPV), and negative predictive value (NPV) of all four first-line agents phenotypic susceptibility testing (DST) and WGS-based antimicrobial resistance (AMR) predictions are shown in Table 1. The isolates that showed discordance were further analyzed to determine the cause of discordance.

### 2.1. Isoniazid

Phenotypic susceptibility testing results of isoniazid at a critical concentration of 0.1 µg/mL by phenotypic DST were correlated with any one mutation found in the *fabG1*, *katG*, *aphC*, or *inhA* genes. The sensitivity, specificity, PPV, and NPV of isoniazid are listed in Table 1. Of the 1510 isolates tested for isoniazid, 1367 (90.5%) were susceptible and 143 (9.5%) were resistant according to phenotypic DST. Of the phenotypically susceptible isolates, 1359 exhibited no mutations, and 8 isolates showed a mutation in the *fabG1*, *katG*, *aphC*, or *inhA* genes, as listed in Table 2. Of these, TB Profiler predicted a susceptible result for three isolates, but remaining five showed the same mutations in TB Profiler as those observed by Mykrobe, i.e., *fabG1*:-15C>T, *katG*:Ser315Thr, *katG*:Ser315Thr, *fabG1*:-17G>T, and *inhA*:Ser94Ala. Of the phenotypically resistant isolates, 124 showed a mutation in either the *fabG1*, *katG*, *aphC*, or *inhA* genes and were correctly predicted to be resistant by *Mykrobe*. The remaining 19 isolates lacked any mutation screened by *Mykrobe*. Further analysis showed that 8 were predicted resistant by TB Profiler (*katG*:-8918_*2727del, *katG*:Trp328Cys, *katG*:-9786_*28940del, *katG*:951_961delCGAGGTCGTAT, *inhA*:Ile21Val, *inhA*:Ile21Val, *katG*:158_171del CCGTCGCTGACCCG + *katG*:152delA, and *fabG1*:-17G>T), while 11 were predicted to be susceptible by TB Profiler. AMR predictions for “Mykrobe corrected values using TB Profiler” slightly improved the statistical prediction values for isoniazid, as shown in Table 1. The two most common codon changes/nucleotide mutations linked to isoniazid resistance were *katG*:S315X (62.9%) and *fabG1* C-15 (33.3%) (these percentages are taken into account when present in combination). Other mutations are listed in Table 2. Isolates with mutations in the *fabG1*:C-15X, *fabG1*:G-17T, *katG*:S315X, *inhA*:S94A, *fabG1*:CTG607CTA, and wild type *fabG1*, *katG*, *aphC*, *inhA* genes showed concordance with the phenotypic susceptibility testing results. 

### 2.2. Rifampin

Out of the 1510 isolates tested for rifampin, 1466 were phenotypically susceptible, and 44 were resistant. The sensitivity, specificity, PPV, and NPV of rifampin are listed in Table 1. Of the susceptible isolates, 1458 (99.5%) revealed no mutation in the *rpoB* gene, but 8 isolates showed an *rpoB* gene mutation in Mykrobe, as shown in Table 3, indicating a discrepancy between the AMR prediction and the phenotypic susceptibility testing results. All isolates that displayed phenotypic resistance exhibited a mutation in the *rpoB* gene, as indicated in Table 3. The isolates with amino acid substitutions at positions 432, 435, 445, and 450 exhibited phenotypic resistance to DST at a 1 µg/mL critical concentration of rifampin. Mykrobe corrected values using TB profiler did not change any statistical predictions for rifampin.

### 2.3. Ethambutol

Ethambutol susceptibility testing was conducted at a critical concentration of 5 µg/m; WGS-based AMR predictions were based on mutations identified in the *embA* and *embB* genes. Of the 1510 isolates tested against ethambutol, 1496 were susceptible, and 14 were resistant; statistical predictions are shown in Table 1. Of the susceptible isolates, 1477 (98.7%) showed no mutation in either the *embA* or *embB* gene. The remaining 19 susceptible isolates exhibited a mutation, indicating a discrepancy between the *Mykrobe* predictions and the phenotypic susceptibilities for ethambutol. Of these 19 discrepant isolates, TB Profiler showed a susceptible result for 3 isolates, resulting in improved PPV by Mykrobe corrected values using TB profiler. Of the 14 phenotypically resistant isolates, all exhibited a mutation in either the *embA* or *embB* gene according to both Mykrobe and TB Profiler. The list of mutations seen in the *embA* and *embB* genes in this study were: *embA*:C-12T, *embB*:M306V, *embB*:M306I, *embB*:M306L, *embB*:G406D, *embB*:G406A, and *embB*:Q497R, as shown in Table 4.

### 2.4. Pyrazinamide

Of the 1431 isolates tested against pyrazinamide, 1341 were phenotypically susceptible, and 90 were resistant. The statistical predictions for pyrazinamide are shown in Table 1. Of the susceptible isolates, 1339 (99.9%) showed no mutation in the *pncA* gene and 2 isolates exhibited a mutation, one each for *pncA*:H57D and *pncA*:P69Q, according to Mykrobe. TB Profiler predicted the former to be resistant and the latter to be susceptible; hence, the Mykrobe corrected values using TB profiler showed a slight improvement in specificity, PPV, and NPV. Of the 90 phenotypically resistant isolates, 43 (47.8%) showed a mutation in the *pncA* gene, while 47 (52.2%) isolates did not possess a mutation, according to *Mykrobe*. Of the 43 resistant isolates with *pncA* mutations, 26 had a *pncA*:H57D, a specific mutation found in *M. bovis*, and 17 isolates exhibited other mutations, as shown in Table 5. Of the 26 *M. bovis*, 19 were identified as *M. bovis* BCG, isolated from urine (*n* = 12) and tissue biopsies (*n* = 7). Of the 47 phenotypically resistant isolates that lacked a known mutation in the *pncA* gene according to the *Mykrobe* database, TB Profiler predicted 38 to be pyrazinamide-susceptible and 9 as pyrazinamide-resistant (*panD*:Ile49Val, *pncA*:Gln10Lys, *pncA*:-11A>C, *pncA*:446_453delATGGCTTG + *pncA*:446_453delATGGCTTG + *pncA*:Arg140Ser, *pncA*:Asp136Asn, *pncA*:386_394delATGTGGTCG, *pncA*:His51Tyr, *pncA*:446_453delATGGCTTG + *pncA*:446_453delATGGCTTG + *pncA*:Arg140Ser, and *pncA*:Leu182Ser); hence, Mykrobe corrected values using TB profiler showed positive impact on sensitivity, PPV, and NPV.

## 3. Discussion

Rapid advancements in DST of MTBC have been achieved through the utilization of molecular predictions of AMR, which have enabled the early detection of antimicrobial resistance. DST for first-line antimicrobials was performed in this study using the CLSI-recommended critical concentrations of these agents and was correlated with WGS-based AMR predictions using *Mykrobe* pipeline [24,26]. Assessment of the sequence quality of WGS showed that the percentage of bases meeting the threshold was high, with a mean of 94.05% and median of 97.87%. The average base coverage was higher than the required 10× for BioHansel identification and AMR detection, with a mean of 82× for our dataset [15,27,28]. For this study WGSs with minimum 30× sequence coverage were included; accordingly, all of the isolates successfully met all pipeline QCs.

Of the 1510 isolates, 1504 were identified using BioHansel, with a majority (93.6%) identified as *M. tuberculosis*, and the remainder belonged to MTBC, which were not *M. tuberculosis*, as mentioned in the results [15]. BioHansel could not identify six MTBC, which revealed incomplete k-mer subtyping issues. By use of a WGS-based SNP analysis developed in our laboratory [25], we were able to successfully identify these six isolates as *M. tuberculosis* L4 (*n* = 1), *M. bovis* (*n* = 2), and *M. africanum* (*n* = 1). Two remaining isolates were found to be *M. tuberculosis* L4, and they both exhibited two distinct SNPs, *gyrB*:C1194T and *gyrB*:G1292C, which had not been observed in any MTBC isolates or lineages in the identification database [25]. The current BioHansel pipeline falls short in identifying these SNPs, and the schema should be revised to include novel markers.

Based on phenotypic testing, we determined that of all isolates tested, 9.5% (143/1510) showed resistance to isoniazid, 2.9% (44/1510) to rifampin, 0.9% (14/1510) to ethambutol, and 6.3% (90/1431) to pyrazinamide. Out of the total isolates, 6.2% (94/1510) showed mono-resistance to isoniazid, 4.3% (61/1431 isolates) showed mono-resistance to pyrazinamide, and 5.2% (8/1510 isolates) showed mono-resistance to rifampin. There was no mono-resistance observed for ethambutol. Canada’s national laboratory-based surveillance data published over the past decade is discordant with the mono-resistance statistics for rifampin and pyrazinamide, with this study overrepresenting mono-resistance statistics. NRCM does not receive all pan-susceptible MTBC isolates for susceptibility testing, as some provincial laboratories conduct phenotypic DST testing onsite but do refer resistant isolates for DST confirmation and WGS to the NRCM [6,7,9]. Isolates in this study were obtained for a 5-year period. Canada’s national laboratory-reported resistance data for all isolates showed that mono-resistant isolates accounted for a total of 8.5%, specifically, 6.6% were isoniazid resistant, 0.3% were RR, 1.6% were pyrazinamide resistant, and as expected, there were no reported isolates of mono-resistance to ethambutol. The report also included 1% MDRs and 0.1% XDRs, and in our study, we determined 1.1% MDR (17/1510) and no XDRs [7]. Of 17 MDRs, 14 were predicted as MDR by both methods, 2 were pre-XDRs according to WGS, and 1 was RR-TB, with no mutation seen for isoniazid. Currently, there is an advantage to performing both genotypic and phenotypic testing. A WHO report on MTBC phenotypic antimicrobial resistance, with data that encompasses results from 45 countries including high and low incidence rates, reported that 35.4% of isolates were resistant to isoniazid, 28.7% to rifampin, 16% to ethambutol, and 14.6% to pyrazinamide [1,4]. These results contrast with those of our study. High rates of TB in countries with endemic TB, differences in testing methods, and inequalities in access to TB diagnosis and treatment may explain the low resistance rates obtained from our study and the high rates documented by the WHO. Our DST AMR rates are more comparable with those from the UK, where it was reported that 7.3% of TB cases were resistant to isoniazid, 1.9% to rifampin, 2% to ethambutol, and 3.2% to pyrazinamide [10]. The similarity between these published rates and the current Canadian subset is likely due to the similar grading of the Healthcare Access and Quality Index from 1990–2016 for UK and Canada, which has shown similar trends of improvement since 1990 [10,29].

In order to predict resistance to isoniazid, the Mykrobe analyzes sequence modifications in the *fabG1*, *katG*, *aphC*, and *inhA* genes, specifically 9, 48,933, 3, and 3, respectively [24]. Table 1 presents the results of the WGS-based AMR predictions for isoniazid, indicating sensitivity, specificity, PPV, and NPV values of 86.71%, 99.41%, 93.94%, and 98.62%, respectively. According to Hunt et al., these statistical predictions for isoniazid were determined to be 94.8%, 99.5%, 94.3%, and 99.6%, while CRyPTIC reported values of 97.1%, 99%, 97.9%, and 98.6%, respectively [21,24]. Interestingly, our study reveals a lower sensitivity in predicting WGS-based AMR for isoniazid. Within our dataset, there were 19 phenotypically isoniazid-resistant isolates that did not contain a mutation present in the *Mykrobe* database. TB Profiler results showed that out of the 19, 11 were predicted to be susceptible, but 8 were predicted to be resistant, with the mutations listed in results. Phenotypic susceptibility testing was repeated for these isolates, and the results were reproducible. Our results support the accuracy of AMR prediction for isoniazid susceptibility using the *fabG1*, *katG*, *aphC*, and *inhA* target mutations, but also suggest that there are additional resistance mutations or other mechanisms of isoniazid resistance not captured in the *Mykrobe* and TB Profiler databases. This study’s PPV for isoniazid was 94% for all mutations, which is consistent with the WHO’s reported PPV of 95.6%. PPV for *fabG1*:G-17T mutations were 50% (*n* = 2), and *inhA*:S94A mutations were 0% (*n* = 1) from our dataset, which were in disagreement with the WHO rates of 86.6% and 85.2% PPV, respectively [19]. As the number of isolates is too low for this genotype, we cannot accurately calculate their PPV. Other studies have shown that isolates harboring a combination of *inhA*:c-15t and *inhA*:S94A or *katG* mutations show moderate- to high-level isoniazid resistance [30]. Combinations of the *inhA* promotor, *inhA*, and *katG* mutations showing high-level isoniazid-resistance were correctly identified in our study [30]. In contrast, a single mutation in the *inhA* promotor or *inhA* gene is associated with low-level isoniazid-resistance [31], and two strains were determined to be phenotypically isoniazid-susceptible in our study. As per the WHO, this *inhA*:S94A mutation is associated with ethionamide resistance (interim), but its significance for isoniazid resistance is not known [19]. Accordingly, our finding, is in line with WHO catalog or alternatively phenotypic DST testing may exhibit technical challenges. This study also observed a *fabG1*:CTG607CTA mutation in our dataset that was not listed in the WHO catalog, but was found to exhibit 100% PPV, with a total of six phenotypically isoniazid-resistant isolates that contained no other mutations for isoniazid genetic markers. To our knowledge, this is a unique mutation, and it requires a larger dataset for characterization. This observation highlights the importance of conducting WGS and phenotypic results validation on local datasets to facilitate correct interpretations within the context of local TB epidemiology, with the potential for new predictive mutation discovery.

The *rpoB* gene was analyzed for 1865 sequence modifications in *Mykrobe* [24]. The sensitivity, specificity, PPV, and NPV of rifampin in this study were 100%, 99.45%, 84.62%, and 100%, respectively (Table 1). This is in comparison to the study by Hunt et al., which showed predictions as 100%, 99.16%, 79.17%, and 100%, compared to 97.5%, 98.8%, 97%, and 99%, published by CRyPTIC [21,24]. Our results are concordant with both of these studies, with the reported PPV of 97% by CRyPTIC being slightly higher than the results in both our study and that of Hunt et al. [24], but lower than that of 70% reported by WHO [19]. CRyPTIC utilized a larger number of isolates and a larger sampling of rifampin-resistant isolates from high-burden countries. The D435V mutation with a PPV of 100% (*n* = 5) in our study and 92.9% by WHO confirms this amino acid as a target for antimicrobial resistance. Also, in our study, a single isolate with an L430P mutation was phenotypically susceptible, with a 50.7% PPV according to WHO [19]. Another isolate with the mutation Q432K was phenotypically resistant, and the WHO PPV for this mutation was 97.1%. Isolates with a unique L452P (*n* = 2) mutation were phenotypically susceptible, but WHO only lists the L452M and L452Q mutations with an associated PPV of 0%, suggesting mutations at this position may carry low or no confidence for rifampin-resistance prediction [19,20]. 

The second most common mutation associated with the *rpoB* gene was at amino acid H445. As per the WHO, the H445C mutation has a PPV of 92.3%, but our data showed that a single isolate was phenotypically susceptible. Additional variants were H445Y (*n* = 7, 6 resistant), 4 with H445D (*n* = 4, all resistant), 1 each of H445N and H445L with a susceptible DST result. The most common mutation observed in our dataset was at amino acid S450. The WHO lists mutations of S450W and S450F with 96.8% and 100% PPV, respectively. Our dataset included 1 isolate with the S450F mutation and 27 isolates with a S450L mutation, all of which were resistant (100% PPV for both). This mutation is associated with high-level resistance to rifampin and cross-resistance to rifabutin [32]. The concordant results for S450 between our dataset and the WHO list, combined with the fact that this was the most common mutation observed among isolates resistant to rifampin, supports the importance of this site as a primary analytical target for AMR prediction. A discrepancy with phenotypic and genotypic results is likely due to low-level resistance to a drug or to technical issues encountered while testing. It should also be noted that this is one of the target regions used by Cepheid in GeneXpert MTB/Rif assays [33].

The *embA* and *embB* genes are analyzed for one of 3 and 27 sequence modifications, respectively, in *Mykrobe* to predict resistance to ethambutol. The ethambutol’s sensitivity, specificity, PPV, and NPV were recorded as 100%, 98.73%, 42.42%, and 100%, respectively, in this study (Table 1); as 98.61%, 98.86%, 59.66%, and 99.98%, respectively, by Hunt et al.; and as 94.6%, 93.6%, 75.1%, and 98.8%, respectively, by CRyPTIC [21,24]. In all three studies, the PPV of ethambutol was low. The presence of *embB* M306* and G406* mutations in our dataset has been found to have a negative impact on the PPV. This finding is unexpected, considering that Hunt et al. [24] reported that the addition of *embB*:M306L to the Mykrobe CP3, which is the final variant panel, enhanced the predictive values. However, in our dataset, the phenotypic resistance was 50% for M306* mutations and 0% for G406* mutations. Of the 19 isolates predicted to be resistant, but found to be phenotypically susceptible, 17 exhibited one of the above *embB* mutations. Other studies have found similar poor PPV results for ethambutol [10,19,24,34]. The WHO mutation catalog lists a poor PPV for all M306 and G406 mutations, with an average of 74.5% and 55.3%, respectively. According to the WHO, the PPV is 83.1% for the mutation M306V, 79.1% for M306L, and 61.2% for M306I. In our dataset, these were 54.5%, 0%, and 44.4%, with a total of 11, 1, and 9 isolates, respectively. These mutations may potentially be associated with resistance at levels lower than or equal to the critical concentration of 5 µg/mL (publication under review), or there may be additional mutations that contribute to resistance, demonstrating a synergistic effect.

The analysis of pyrazinamide resistance predictions utilizing the *pncA* gene involves examining 1 out of 12,812 sequence modifications in *Mykrobe*. The sensitivity, specificity, PPV, and NPV of pyrazinamide were 47.78%, 99.85%, 95.56%, and 96.61%, respectively, in this study; 81.6%, 99.43%, 81.6%, and 99.43%, respectively, as per Hunt et al.; and 91.3%, 96.8%, 80.9%, and 98.7%, respectively, as per CRyPTIC [21,24]. The sensitivity for pyrazinamide from our dataset is lower than that in both studies and may be explained by a mutation in the *pncA* gene present in our Canadian dataset, which is not included in the *Mykrobe* database (e.g., an 8 bp deletion at position 446 and an Arg140Ser frameshift mutation) [35,36]. Pyrazinamide showed the lowest sensitivity, at only 47.78%, but the PPV was 95.56%. Of the isolates predicted to be resistant by *Mykrobe*, all mutations were in the *pncA* gene, with a total of 17 different mutations being identified. Of these, only *pncA*:P69Q was susceptible phenotypically, but a PPV cannot be calculated, as there was a single isolate with this mutation. The majority of isolates (26 of 43 resistant) exhibited a *pncA*:H57D mutation known to cause resistance in *M. bovis* isolates, with a calculated PPV of 96.3%, correlating with the results from the WHO, which lists a PPV of 98.1% [19]. All other mutations were found in only one or two isolates; these numbers are too small to accurately determine the PPV for each mutation. Interestingly, our dataset included isolates with mutations not listed in the WHO mutation catalogue: *pncA*:TX192TA and *pncA*:TCG490TAG. Due to each mutation being seen only once, we cannot determine their role in pyrazinamide resistance at this time. 

When there is a discrepancy between two methods, i.e., phenotypic DST and WGS-based AMR predictions by Mykrobe, integration of a second pipeline TB Profiler can be helpful. Mykrobe corrections using TB profiler improved statistical predictions for isoniazid, ethambutol, and pyrazinamide, but rifampin predictions remained unchanged. The mutation databases for both pipelines need to be updated constantly, and there is a necessity for novel mutations to be included into AMR prediction databases and tools. Recently, the WHO has launched recommendations regarding the use of targeted next-generation sequencing for drug-resistant tuberculosis diagnosis [37]. It is also worth noting that phenotypic DST critical concentrations for MTBC have not been updated in some time, and MTBC remains one of the few pathogens that are tested using critical concentration rather than minimum inhibitory concentrations (MICs). Revisions of clinical outcomes at different testing concentrations should be undertaken to determine whether a change in critical concentration is needed, or whether a shift to MIC-based testing should be considered, which may have an impact on WGS-phenotypic test correlation.

A high-quality molecular assay should demonstrate a minimum sensitivity of 90% and more than 95% specificity [20,38]. These targets were met for rifampin and ethambutol from our dataset; however, the PPV for ethambutol was lower than this level. Isoniazid showed a calculated sensitivity of 86.71%, which is just below the WHO threshold. In our dataset, this sensitivity was slightly improved by supplementing TB Profiler results, as this was able to resolve some of these discrepancies, with a few isolates showing reproducible results using both pipelines, indicating mutation database impacts on AMR predictions. Pyrazinamide had a low sensitivity of 47.78%, which falls far below the WHO recommendations, but this may be due to unique resistance mutations which are not yet included in the current version of *Mykrobe*. The specificity for all first-line agents was >98%, according to by both *Mykrobe* and TB Profiler, indicating that these databases had a low false-positive prediction rate. It is known that the pipelines utilized to identify or predict resistance are limited by the amount of genomic information that they contain. As more isolates are characterized worldwide and new mutations are discovered, with direct or indirect linkages to resistance or susceptibility, they should be included in bioinformatics pipelines, with their statistical predictions and confidence values. 

This study conducted a retrospective analysis on the bioinformatic pipelines and algorithms utilized within our laboratory and AMR prediction statistics relevant to Canada. The findings of this study demonstrate that WGS-based AMR testing exhibits a strong correlation with phenotypic testing, but that the accuracy of the results is impacted by the database of mutations used to predict resistance. With the increasing use of genomics for AMR and the continual revision of these AMR databases and pipelines to include new validated mutations, their use in routine diagnostics for susceptibility testing is becoming common. It is important to routinely update these databases and incorporate them into easy-to-use software that does not require an understanding of command-line interface. Although phenotypic antimicrobial susceptibility is the gold standard, both assays used in conjunction are important in AMR determination. WGS-based or molecular assays have the potential to decrease testing costs, providing rapid identification, AMR, and genotyping results with use of a single test. Furthermore, they can aid in faster initiation of appropriate treatment regimens for resistant TB cases, thereby contributing to the global effort to manage TB disease.

In conclusion, our results show that there is a strong correlation between phenotypic anti-tuberculous first-line drug susceptibility and whole-genome sequence-based antimicrobial susceptibility prediction. However, there needs to be a focus on improving the predictions of ethambutol and pyrazinamide susceptibility, and prediction databases need to be updated with the latest high-confidence mutations to reflect the evolving understanding of the genetic determinants of antimicrobial resistance.

## 4. Materials and Methods

### 4.1. Selection of Strains

Using the mycobacterial database at the NRCM of the National Microbiology Laboratory (NML), 1510 isolates belonging to the MTBC were included in this study. Some provinces may submit all TB cultures for testing to the NRCM, while others may not. The selection criteria included isolates that underwent WGS between the years 2018 to 2022, underwent DST, and yielded WGS data that met the specified sequence quality. The acceptable sequence quality was defined as a minimum WGS sequence coverage depth of 30× aligning to the *M. tuberculosis* H37Rv reference genome NC_000962.3. WGS was performed by the NRCM, while phenotypic DST for first-line agents was performed either by the NRCM or the provincial TB reference laboratories: Shared Health, Manitoba; Regina Qu’Appelle Health Region, Saskatchewan; Public Health Laboratory, B.C. Center for Disease Control, BC; LSPQ—Institut National de Santé Publique du Québec, Québec; and Alberta Precision Laboratories—Public Health, Alberta. 

### 4.2. First-Line Antimicrobial Susceptibility Testing

First-line antimicrobial susceptibility testing on all MTBC isolates was performed using Bactec 960, as per the manufacturer’s instructions (Becton Dickinson, Mississauga, ON, Canada). The recommended critical concentrations of isoniazid at 0.1 µg/mL, rifampin at 1 µg/mL, ethambutol at 5 µg/mL, and pyrazinamide at 100 µg/mL were tested [26,39]. The susceptibility testing was repeated for confirmation when genotypic and phenotypic results showed discordance. The culture purity was tested on blood agar and Middlebrook 7H10 agar plates. Phenotypic DST data for isoniazid, rifampin, and ethambutol were available for all 1500 isolates. For pyrazinamide, phenotypic DST data were available for 1431 isolates, as one laboratory does not routinely perform pyrazinamide phenotypic testing.

QC was performed in tandem with each susceptibility assay and for the new antimicrobial lot using *M. tuberculosis* H37Rv ATCC 27294. When an isolate was resistant to one or more of the antimicrobials, DST was systematically repeated in parallel with the resistant QC strain to confirm resistance. The QC strains used were *M. tuberculosis* ATCC 35822 (resistant to isoniazid), *M. tuberculosis* ATCC 35838 (resistant to rifampin), *M. tuberculosis M. tuberculosis* ATCC 35837 (resistant to ethambutol), and *M. tuberculosis* ATCC 35828 (resistant to pyrazinamide). 

### 4.3. DNA Extraction and Whole Genome Sequencing

Genomic DNA extraction for WGS was adapted from Shea et al. [40]. Briefly, DNA was extracted from either 1–2 mL of MTBC-positive MGIT^TM^ media or a loopful of culture suspended in 1 mL of sterile water. Cultures were then centrifuged for 20 min at 4 °C at 4000× *g*. Bacterial pellets were gently suspended in 250 µL of InstaGene™ Matrix (Bio-Rad, Mississauga, ON, Canada), transferred to a screw-capped tube containing 0.5 mm zirconium beads, and incubated at 56 °C for 15 min [40]. The tubes were submerged in boiling water for 20 min. Bead beating was performed using a Fast-Prep 24 homogenizer for two cycles of 45 s at 4.5 m/s. The tubes were centrifuged at 18,000× *g* at 4 °C for 20 min to pellet the InstaGene Matrix and cell debris. The supernatant was stored at 4 °C until submitted for WGS. Quantification of genomic DNA was performed using the Qubit™ 1X dsDNA HS Assay Kit and the Qubit 3 fluorometer (Thermo Fisher Scientific, Waltham, MA, USA).

WGS was performed on an Illumina MiSeq platform (Illumina Inc., San Diego, CA, USA), using either the MiSeq Reagent Kit v2 (300-cycle) or the MiSeq Reagent Kit v3 (600-cycle) by the Genomics Core Facility at the NML. The Integrated Rapid Infectious Disease Analysis (IRIDA) platform served as the storage location for the WGS data. IRIDA is a web-based and secure platform that possesses the ability to integrate WGS, laboratory metadata such as phenotypic DST data, submitter information, and bioinformatic pipeline outputs, which helps in conducting a thorough genomic epidemiological analysis [41].

### 4.4. Data Analyses

Galaxy (version 20.01) is an open-source and web-based platform for bioinformatic analyses [42]. Sequence QC reports using FastQC (Galaxy version 0.72+galaxy1) and MultiQC (Galaxy version 1.11+galaxy1) were generated for each WGS, using the *M. tuberculosis* H37Rv NC_000962.3 as a reference sequence [43]. WGSs with a minimum sequence coverage depth of 30× were used in this study [15,24,27,28,43].

The purity of the extracted DNA utilized for WGS was verified, and the organisms were classified by employing the Kraken2 tool in Galaxy, which provided a comprehensive list of contaminating bacteria, mycobacteria, MTBC, and eukaryotic sequence matches [14]. To identify MTBC isolates in this study, we used BioHansel workflow, developed in our laboratory, to look for specific mutations on 33 bp k-mer tiles that have been associated with various MTBC species [15]. This workflow can identify all established species of the MTBC and its rare non-validated species [25].

Galaxy workflow *Mykrobe Predictor* v0.7.0 and v0.10.0 were used for predicting AMR to isoniazid, rifampin, ethambutol, and pyrazinamide, based on known resistance mutations [14]. AMR predictions as susceptible or resistant were determined based on 21 bp k-mer tiles, targeting specific regions of the MTBC genome [16,24]. WGS-based AMR predictions for isoniazid in *Mykrobe* are based on the *fabG1* gene (744 nucleotides plus a 140 nucleotide upstream region), encoding 247 amino acids, the *katG* gene (2223 nucleotides plus a 37 nucleotide upstream region), encoding 740 amino acids, the *aphC* gene (588 nucleotides plus a potential 105 nucleotide upstream region), encoding 195 amino acids, and the *inhA* gene (810 nucleotides plus an 18 nucleotide upstream region), encoding 269 amino acids. The *rpoB* gene consists of 3519 nucleotides, along with a 497 nucleotide upstream region encoding 1172 amino acids, and it is used to predict AMR for rifampin. The *embA* gene (285 nucleotides plus an 85 nucleotide upstream region) and the *embB* gene (3297 nucleotides) is used to predict AMR for ethambutol. The *pncA* gene spans a length of 565 nucleotides, with an additional 40 nucleotides upstream, and it is used to detect WGS-based AMR for pyrazinamide [24].

The *Mykrobe* Parser 202010 panel was used to combine several outputs from *Mykrobe* workflow into line data [44]. The TB Profiler v4.4.1 workflow was used to look for additional mutations; for example, this pipeline was run on isolates that showed phenotypic resistance to an agent and a susceptible *Mykrobe* AMR prediction or phenotypic susceptible isolates that showed a resistant *Mykrobe* AMR prediction [13]. These values were referred to as “Mykrobe corrections using TB profiler” (Table 1).

### 4.5. Statistical Analysis

The statistical prediction values of sensitivity, specificity, PPV, and NPV were calculated based on phenotypic testing, as the gold standard, and WGS-based AMR prediction, as the test method [45]. Sensitivity was calculated as the number of specimens predicted to be resistant divided by the total number of specimens phenotypically resistant. Specificity was calculated as the number of specimens predicted to be susceptible divided by the total number of specimens phenotypically susceptible. PPV was calculated as the number of truly resistant specimens divided by the number of predicted resistant specimens. NPV was calculated as the number of truly susceptible specimens divided by the number of predicted susceptible specimens.

## Figures and Tables

**Table 1:** The predictive values (positive predictive value (PPV), and negative predictive value (NPV), sensitivity, and specificity) of AMR predictions for the four primary antituberculosis antimicrobials.

Antimicrobial	Pipeline Used	Total with Phenotypic and Genotypic Results	Phenotypic Resistance		Phenotypic Susceptible		Sensitivity	Specificity	PPV	NPV
			WGS Predicted Resistant	WGS Predicted Susceptible	WGS Predicted Resistant	WGS Predicted Susceptible				
Isoniazid	Mykrobe	1510	124	19	8	1359	86.71%	99.41%	93.94%	98.62%
	Mykrobe corrected with TB Profiler *	1510	132	11	5	1362	92.31%	99.63%	96.35%	99.20%
Rifampin	Mykrobe	1510	44	0	8	1458	100.00%	99.45%	84.62%	100.00%
	Mykrobe corrected with TB Profiler *	1510	44	0	8	1458	100.00%	99.45%	84.62%	100.00%
Ethambutol	Mykrobe	1510	14	0	19	1477	100.00%	98.73%	42.42%	100.00%
	Mykrobe corrected with TB Profiler *	1510	14	0	16	1480	100.00%	98.93%	46.67%	100.00%
Pyrazinamide	Mykrobe	1431	43	47	2	1339	47.78%	99.85%	95.56%	96.61%
	Mykrobe corrected with TB Profiler *	1431	52	38	1	1340	57.78%	99.93%	98.11%	97.24%

* Mykrobe corrected with TB Profiler: any result that was discrepant between phenotypic phenotypic susceptibility testing (DST), and Mykrobe AMR prediction in our laboratory was further tested using TB profiler. If the discrepancy was resolved using TB profiler, the prediction value was corrected.

**Table 2:** Phenotypic susceptibility testing results for isoniazid, and mutations found in *fabG1*, *katG*, *aphC*, and *inhA* genes with WGS-based AMR predictions using Mykrobe v0.7.0.

Gene:Nucleotide/Codon Change	Phenotypic Resistant	Phenotypic Susceptible	WGS-Based Isoniazid Prediction
*fabG1*:G-17T	1	1	R
*fabG1*:C-15X	30	1	R
*fabG1*:T-8X + *katG*:S315X	2	0	R
*fabG1*:C-15X + *katG*:S315X	4	0	R
*fabG1*:C-15X + *aphC*:G-48A	1	0	R
*fabG1*:C-15X + *inhA*:I194T	5	0	R
*fabG1*:C-15X + *inhA*:I21T	1	0	R
*fabG1*:C-15X + *inhA*:S94A	2	0	R
*fabG1*:CTG607CTA	6	0	R
*katG*:S315X	72	5	R
*inhA*:S94A	0	1	R
*fabG1*, *katG*, *aphC*, and *inhA:* WT*	19	1359	S

WT*: Wild type refers to a non-mutated gene of the reference strain.

**Table 3:** Results of phenotypic susceptibility testing results for rifampin, and mutations found in the *rpoB* gene with WGS-based AMR predictions using Mykrobe.

Gene:Codon Change	Phenotypic Resistant	Phenotypic Susceptible	WGS-Based RIF Predictions
*rpoB*:L430X	0	1	R
*rpoB*:Q432X	1	0	R
*rpoB*:D435X	5	0	R
*rpoB*:H445X	10	4	R
*rpoB*:S450X	28	0	R
*rpoB*:L452X	0	2	R
*rpoB*:I491F	0	1	R
*rpoB*: WT*	0	1458	S

WT*: Wild type refers to a non-mutated gene of the reference strain.

**Table 4:** Phenotypic susceptibility testing results for ethambutol, and mutations found in *embA* and *embB* genes with WGS-based AMR predictions using Mykrobe.

Gene:Nucleotide/Codon Change	Phenotypic Resistant	Phenotypic Susceptible	WGS-Based Ethambutol Prediction
*embB*:M306V	6	5	R
*embB*:M306I	4	5	R
*embB*:M306L	0	1	R
*embB*:G406D	0	5	R
*embB*:G406A	0	1	R
*embB*:Q497R	3	1	R
*embA*:C-12T	0	1	R
*embA*:C-12T + *embB*:M306V	1	0	R
*embAB*: WT*	0	1477	S

WT*: Wild type refers to a non-mutated gene of the reference strain.

**Table 5:** Phenotypic susceptibility testing results for pyrazinamide, and mutations found in the *pncA* gene with WGS-based AMR predictions using Mykrobe.

Gene:Mutation	Phenotypic Resistant	Phenotypic Susceptible	WGS-Based Prediction
*pncA*:A-11G	1	0	R
*pncA*:L27P	1	0	R
*pncA*:D49N	1	0	R
*pncA*:H51Y	1	0	R
*pncA*:H57D	26	1	R
*pncA*:W68R	1	0	R
*pncA*:P69Q	0	1	R
*pncA*:S104R	1	0	R
*pncA*:G108R	1	0	R
*pncA*:G132A	2	0	R
*pncA*:GCA136GCG	2	0	R
*pncA*:V139G	1	0	R
*pncA*:A146E	1	0	R
*pncA*:L151S	1	0	R
*pncA*:V180G	1	0	R
*pncA*:T192TA	1	0	R
*pncA*:TCG490TAG	1	0	R
*pncA*: WT*	47	1339	S

WT*: Wild type refers to a non-mutated gene of the reference strain.

## Data Availability

Due to data sharing restrictions from multiple government organizations, this data cannot be publicly shared at this time.
